# Possible Secondary Population-Level Effects of Selective Harvest of Adult Male Muskoxen

**DOI:** 10.1371/journal.pone.0067493

**Published:** 2013-06-20

**Authors:** Joshua H. Schmidt, Tony S. Gorn

**Affiliations:** 1 U.S. National Park Service, Central Alaska Network, Fairbanks, Alaska, United States of America; 2 Division of Wildlife Conservation, Alaska Department of Fish and Game, Nome, Alaska, United States of America; University of Tasmania, Australia

## Abstract

Selective harvest regimes are often focused on males resulting in skewed sex-ratios, and for many ungulate species this strategy is sustainable. However, muskoxen (*Ovibos moschatus*) are very social and mature bulls (≥4 years old), particularly prime-age bulls (6–10 years old), play important roles in predator defense and recruitment. A year-round social structure incorporating large males into mixed-sex groups could make this species more susceptible to the effects of selective harvest if population composition and sex-ratios influence overall survival and reproductive success. Using detailed data collected on the muskox population occupying the Seward Peninsula, Alaska during 2002–2012, we formulated the hypothesis that the selective harvest of mature bulls may be related to documented changes in population composition and growth rates in this species. In addition, we reviewed existing published information from two other populations in Alaska, the Cape Thompson and Northeastern populations, to compare population growth rates among the three areas under differential harvest rates relative to our hypothesis. We found that on the Seward Peninsula, mature bull:adult cow ratios declined 4–12%/year and short-yearling:adult cow ratios (i.e., recruitment) declined 8–9%/year in the most heavily harvested areas. Growth rates in all 3 populations decreased disproportionately after increases in the number of bulls harvested, and calf:cow ratios declined in the Northeastern population as harvest increased. While lack of appropriate data prevented us from excluding other potential causes such as density dependent effects and changes in predator densities, our results did align with our hypothesis, suggesting that in the interest of conservation, harvest of mature males should be restricted until causal factors can be more definitively identified. If confirmed by additional research, our findings would have important implications for harvest management and conservation of muskoxen and other ungulate species with similar life-histories.

## Introduction

Ungulate harvest regimes are often selectively focused on males with the goal of increasing total sustainable harvest [1] and providing increased trophy value. These strategies frequently result in skewed sex and age ratios at the population level [2]. Research on cervid species including moose (*Alces alces*) [3], [4] and mule deer (*Odocoileus hemionus*) [5], [6] has found little evidence to suggest that these reductions affect productivity, despite large changes to overall population composition. However, exceedingly female-biased sex ratios can have long term demographic and genetic effects on populations [7], [8], [9], [10], [11], and in some circumstances these effects can lead to declines in reproductive success [2], [8], [12] or calf survival and recruitment [7], [13], [14]. These types of secondary effects are difficult to detect but can have major implications for the long term sustainability of harvested populations.

In contrast to many ungulate species, muskoxen (*Ovibos moschatus*) are quite gregarious and form persistent mixed-sex and age groups throughout the year, although a portion of the males in the population occur in smaller bachelor groups [15], [16]. Bulls are considered to be mature at 4 years of age, although they do not attain maximum body mass until they reach approximately 6 years of age [17]. This delayed growth pattern corresponds with observations that the majority of harem bulls in unharvested populations are between 6 and 10 yrs old [17], [18] (hereafter: ‘prime-age’ bulls), resulting in a relatively small number of prime-aged individuals being responsible for most of the breeding [17]. The group-living social structure of muskoxen has been shown to be important for both predator defense [15] and other activities such as foraging [19]. Although females will charge predators [20], mature bulls frequently play a lead role in defending the group [21]. Similar defensive strategies have been observed in other group-living species such as wood bison (*Bison bison athabascae*) [22]. Mature bull muskoxen in general, and prime-aged bulls in particular, often place themselves between the perceived threat and the rest of the group and increase group cohesion during attacks [15], [17], [23], [24], [25], [26]. Due to their larger size (cows are approximately 40–50% smaller than prime-aged bulls [15], [26]), they may be more able to successfully defend against predators, and even if killed during an attack, the remaining group members may escape unharmed. When larger numbers of these individuals occur in a population, survival rates for cows and calves may be increased. These important breeding and leadership functions suggest that the presence of prime-aged bulls could influence group-level survival and productivity throughout the year.

Research on similar species, such as Cape buffalo (*Syncerus caffer caffer*) and wood bison, also suggests that bachelor groups and higher male:female ratios may perform critical functions by allowing breeding bulls an opportunity to recover during extended reproductive seasons [27], [28]. Without a large pool of available prime-aged males, the breeding period could be extended or less successful, and males may experience higher mortality rates due to decreased body condition. When social, reproductive, and defensive roles are considered together, the importance of prime-aged males could make muskoxen and other ungulate species with similar life history strategies much more sensitive to selective harvest of mature males.

Muskoxen formerly occurred throughout much of the Canadian Arctic, Greenland, and northern Alaska, but by the mid to late 1800s muskoxen were absent from Alaska [26], [29], and populations in Canada were greatly reduced [15]. Since then, muskoxen have been successfully reintroduced to Alaska, and populations have recovered across much of Canada. However, despite low apparent harvest rates (e.g., 1–6%), population growth rates in the 3 mainland populations in Alaska (i.e., the Seward Peninsula population [SPP], the Northeastern population [NEP], and the Cape Thompson population [CTP]) have all declined over time [30], [31], [32]. Many potential causes for these changes in population growth have been identified including: density dependence, harsh winter weather and disease [33], [34], and increased predation and emigration [33], [35]. Interestingly, these declines in population growth rates also occurred after increases in harvest, suggesting that the effects of the selective harvest regimes should be more closely considered as a potential driver as well. However, because basic population metrics and biological information for muskox are lacking in many areas, it can be difficult to identify the primary causal factors related to population trajectory.

Historically, the males-only harvest regimes in Alaska were concentrated on mature bulls (≥4 years of age) due to their higher trophy value and difficulties in distinguishing immature males from females. The tendency for mature bulls, particularly prime-aged individuals, to place themselves between the rest of the group and any perceived threat [21] may have further increased harvest pressure on this segment of the population. Although some basic biological information is lacking, the differences in social structure relative to many other ungulates, the potential for high relative harvest rates of prime-aged males, and the apparent similarities in population trajectories relative to harvest among the 3 Alaska mainland populations led us to formulate the hypothesis that selective harvest of mature bulls may have secondary population-level impacts at the group level, possibly through changes in survival and recruitment rates, leading to subsequent overall population declines. We used abundance and composition survey data from the SPP to estimate the size of the mature bull and yearling components of the population, realized harvest rates (number of mature bulls harvested/estimated number of mature bulls in the population), and recruitment rates (number of short-yearlings) between 2002 and 2012. We also compared population growth rates, harvest rates, and trends in population composition (where data were available) among the 3 mainland populations of muskoxen in Alaska to identify any patterns relative to our hypothesis. Our primary objectives were to: 1) investigate patterns in population composition and growth rates relative to changes in harvest in the SPP; 2) generate a working hypothesis identifying potential mechanisms for secondary impacts of harvest; 3) identify similarities in harvest rates and population trajectories among 3 harvested populations of muskox in Alaska (i.e., the SPP, NEP, CTP); and 4) provide conservative harvest recommendations and suggest further research needed to establish the causal factors of observed population declines.

## Materials and Methods

### Ethics Statement

This project falls under the definition of a field study as defined by the Animal Welfare Act Regulations§1.1: “Field study means a study conducted on free-living wild animals in their natural habitat. However this term excludes any study that involves invasive procedure, harms, or materially alters the behavior of an animal under study.” Our sampling methods were based solely on visual observations from a distance, were non-invasive, and did not harm or materially alter the behavior of the animals observed in this study. Under §2.31, d,1 of the Animal Welfare Act Regulations, field studies are exempt from IACUC review. Because this project met the definition of a ‘field study’ as defined by the Animal Welfare Act Regulations, a permit was not required. This project also complied with the U.S. National Park Service Planning, Environment, and Public Comment (PEPC) process (PEPC Project ID: 41681).

### Study Area

**Figure 1:**
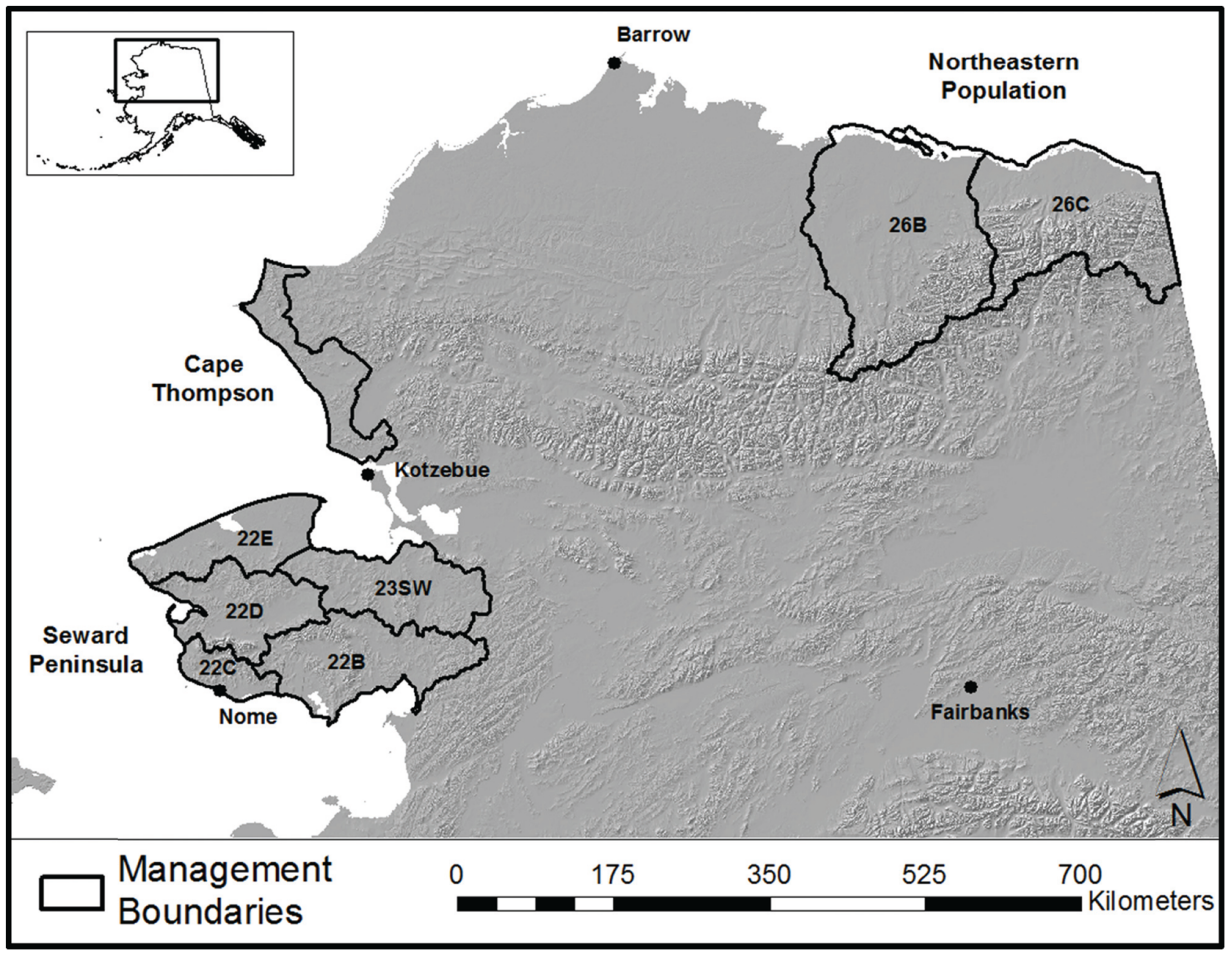
Approximate extent of the 3 Alaska mainland populations of muskoxen. Muskoxen survey and management areas showing the approximate boundaries of each of the 3 Alaska mainland populations (Northeastern, Cape Thompson, and Seward Peninsula) and the relevant Game Management Subunits. Muskoxen in the Northeastern population generally occurred north of the mountains, and small numbers occurred in adjacent areas to the east and west of the delineated boundaries.

The SPP study area consisted of 5 administrative Game Management Subunits ([GMSUs]; 22B, C, D, E, 23SW) covering 65,232 km^2^ of the Seward Peninsula in western Alaska (Fig. 1). For management purposes, all harvest regulations were established at the level of the individual GMSU, as were data collection protocols. The terrain varied from rugged mountains and river valleys to flat coastal wetlands. Spruce forests (*Picea sp.*) occurred in the eastern portions of the SPP study area, while more western areas were treeless and largely tundra covered with willow (*Salix spp.*) thickets along the riparian corridors. During snow free months access to most of the study area is limited, except along the Nome road system in the central Seward Peninsula where almost 645 km of gravel roads can provide hunters access to portions of 22B, 22C, and 22D. Mean monthly temperatures in Nome (in GMSU 22C) vary between −19.3°C and 14.1°C, and average annual snow depth is 158 cm [36]. The NEP survey area consisted of portions of 3 GMSUs (26A, B, C) along the north slope of the Brooks Range in the northeastern portion of Alaska (Fig. 1; see [33], [35] for a detailed description). The CTP survey area consisted of a 10,440 km^2^ portion of GMSU 23 north of Kotzebue, Alaska encompassing Cape Kruzenstern National Monument and the coastal areas north to Cape Thompson (Fig. 1; see [32], [37] for a detailed description).

### Seward Peninsula Population

#### Population Surveys

Abundance estimates between 1983 and 2007 were based on full coverage, minimum count population surveys conducted at regular intervals (i.e., 1983–1985, 1988, 1992, 1994, 1996, 1998, 2000, 2002, 2005, 2007) throughout the SPP study area during spring (generally March and April) when snow coverage was nearly complete and sightability was high [31], [38]. Fixed-wing aircraft (e.g., Piper PA-18, Aviat Husky, Cessna 185) were used to cover all known muskoxen habitat at approximately 3.2–4.8 km intervals. Although pilots were allowed to vary search intensity based on knowledge of the survey area and habitat quality, full coverage was required. During 2010 and 2012, transects were established systematically at 4.8 km intervals throughout the entire study area and estimates of abundance were generated using distance sampling theory [31], [39]. The new survey method was implemented primarily to reduce cost of future surveys and increase the reliability of abundance estimates, while secondarily providing an opportunity to assess potential bias in the minimum counts due to incomplete detection. For analysis, during years when abundance surveys were not conducted, we assumed the GMSU-specific populations grew at a constant rate during the interval between surveys.

#### Sex and Age Composition Surveys

We conducted composition surveys during March and April, prior to calving, within ≥1 GMSUs in most years between 2002 and 2012. Each GMSU was surveyed ≥3 times between 2002 and 2011, and in 2012 all 5 GMSUs were surveyed. Locations of muskox groups were recorded during the peninsula-wide population survey or during pre-composition surveys designed to locate the majority of the groups within the GMSU of interest. We then randomized this list of known groups and sampled them in order until approximately 15 groups or 200 individuals had been sampled within the GMSU of interest. This was consistent with the sample size recommendations for composition surveys proposed by Czaplewski et al. [40], although we did not conduct a formal power analysis to assess the adequacy of sample size. We used a helicopter (Robinson R44) to land near groups and classified each individual into 1 of 5 sex and age categories: mature bulls (≥4 yrs old), immature bulls (2–3 yrs old), mature cows (≥3 yrs old), immature cows (2 yrs old), and short-yearlings (<1 yrs old). Sex and age categories were based on horn development and body size and are highly reliable when assessed by experienced observers [41]. Bulls >4 yrs of age cannot be reliably differentiated, hence all bulls ≥4 yrs of age were considered to be mature. Population abundance and composition data can be found in [31].

#### Sex and Age Composition Estimates

We estimated composition (i.e., sex and age ratios) within each GMSU using an individual based estimator, adjusted for estimated GMSU-specific abundance. Because population and composition surveys were often conducted during different years and group sizes fluctuated annually, the number of groups in each sub-population was unknown. This prevented us from using a group based estimator, possibly introducing some bias due to correlation among individuals within groups [42]. We minimized this risk by sampling randomly from all known groups and observing a relatively large proportion of groups and individuals within the sub-population in each unit. Treating the individual animal as the sample unit allowed us to use GMSU-specific abundance point estimates (interpolated between survey years) as a finite population correction factor. Because harvest regulations and composition surveys were GMSU-specific, all analyses except overall trends in abundance were conducted at the level of the individual GMSU.

We conducted composition analyses in a Bayesian framework using a data augmentation approach [43], although in our case, we were able to limit the possible number of individuals in each sex and age class remaining in the sub-unit. We used a multinomial distribution with 3 categories (mature bulls, mature cows, and short-yearlings) to estimate the probability of each individual belonging to one of these sex and age classes. Data were arranged in matrix format with the number of rows equal to the population estimate and 3 columns, one for each sex and age category. The appropriate category for each observed individual was identified using a 1, with the remaining categories coded as 0s. For example, a mature bull would be coded as 1 0 0, whereas a short-yearling would be coded 0 0 1. Observed animals that did not belong to any of these 3 categories (e.g., an immature cow) were coded as 0 0 0 to indicate that they had been observed but did not belong to any of the main categories of interest. The sex and age of the remaining portion of the estimated number of individuals not included in the composition sample was considered to be unknown. These unknown values were then estimated during each update of the sampler. We estimated ratios for each year and GMSU separately, and mature bull:mature cow and short-yearling:mature cow ratios were calculated during each update of the sampler. We then estimated trends in composition by fitting a generalized linear trend model to a set of 2500 samples from the posterior distributions for the annual composition estimates within each GMSU. This allowed us to estimate rates of change in mature bull:mature cow and short-yearling:mature cow ratios with measures of precision over the study period. The upper and lower 2.5% of estimates were discarded to provide an estimate of the 95% credible interval for each trend. All estimation was conducted with R 2.13.1 [44] and WinBUGS 1.4.3 [45].

#### Harvest Monitoring

Harvest regulations were applied at the level of the individual GMSU and varied among GMSUs annually. All hunters were required to submit a harvest report upon harvesting a muskox or at the end of the season if unsuccessful, and although age of the animal was not consistently recorded, most hunts were limited to bulls only. Because immature bulls and cows can be difficult for inexperienced hunters to distinguish, mature bulls were usually selected to avoid accidentally harvesting a cow. Large bulls were also preferred for their trophy value even though hunters from outside the local area were required to submit the skulls to the Alaska Department of Fish and Game [ADFG] for trophy destruction (i.e., the distal end of each horn was removed and retained) in most hunts. Based on these combined circumstances, we assumed that most bulls harvested from the SPP were mature animals. We assessed the validity of this assumption by calculating the proportion of males ≥4 yrs vs. <4 yrs from a sample (n  =  42) of horns submitted to ADFG for trophy destruction in 2010.

#### Mature Bull and Short-Yearling Abundance and Realized Harvest Rates

We applied the mature bull and short-yearling ratio estimates to the abundance estimate for each year in each GMSU to estimate the abundance of mature bulls and short-yearlings in each hunt unit. Because we were confident that harvest consisted almost entirely of mature males, directly estimating the number of individuals in this subgroup allowed us to calculate the maximum realized harvest rate on this segment of the population. We calculated harvest rate within WinBUGS allowing us to directly provide estimates of precision on the number of mature bulls removed as well as the realized harvest rates. We also estimated the number of short-yearlings in each GMSU in each survey year in the same manner, providing an estimate of recruitment into the population for each survey year. These estimates were only calculated for years in which composition surveys were conducted. Estimates are presented as means with 95% Bayesian credible intervals (CI). Trends in bull and short-yearling abundance were estimated using the approach described above.

### Comparisons Among Populations

We used published minimum count and harvest data from the SPP [31], NEP [33], [35], [46] and CTP [32], [47] to estimate exponential rates of population change during time periods with differing levels of harvest. We grouped years into time periods corresponding to changes in harvest regulations and reported harvest. We then calculated average harvest as a percent of the total population and the average number of bulls harvested during each period. We then compared harvest rates to population growth rates among the three populations to identify similarities. We also compared changes in calf production [33], [35] to harvest over these same time periods for the NEP.

## Results

### Seward Peninsula Population

**Figure 2:**
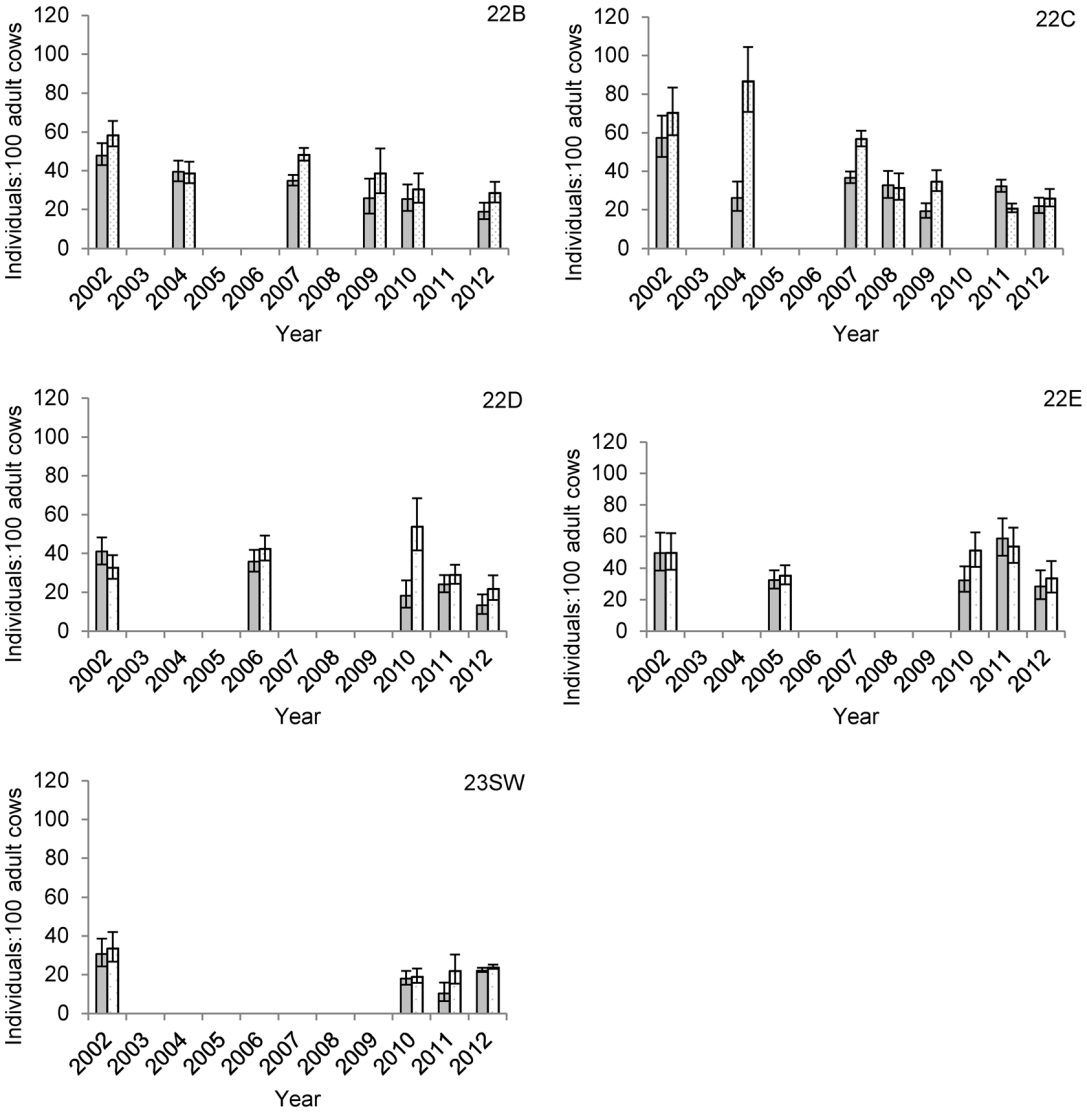
Composition estimates for muskoxen on the Seward Peninsula, Alaska from 2002–2012. Estimates of muskoxen composition for 5 Game Management Subunits (GMSUs) on the Seward Peninsula, Alaska, USA from 2002 to 2012. Gray bars and stippled bars represent short-yearlings:100 adult cows and mature bulls:100 adult cows, respectively. Missing bars indicate years when composition surveys were not completed in a given GMSU. Error bars represent 95% Bayesian credible intervals.

**Figure 3:**
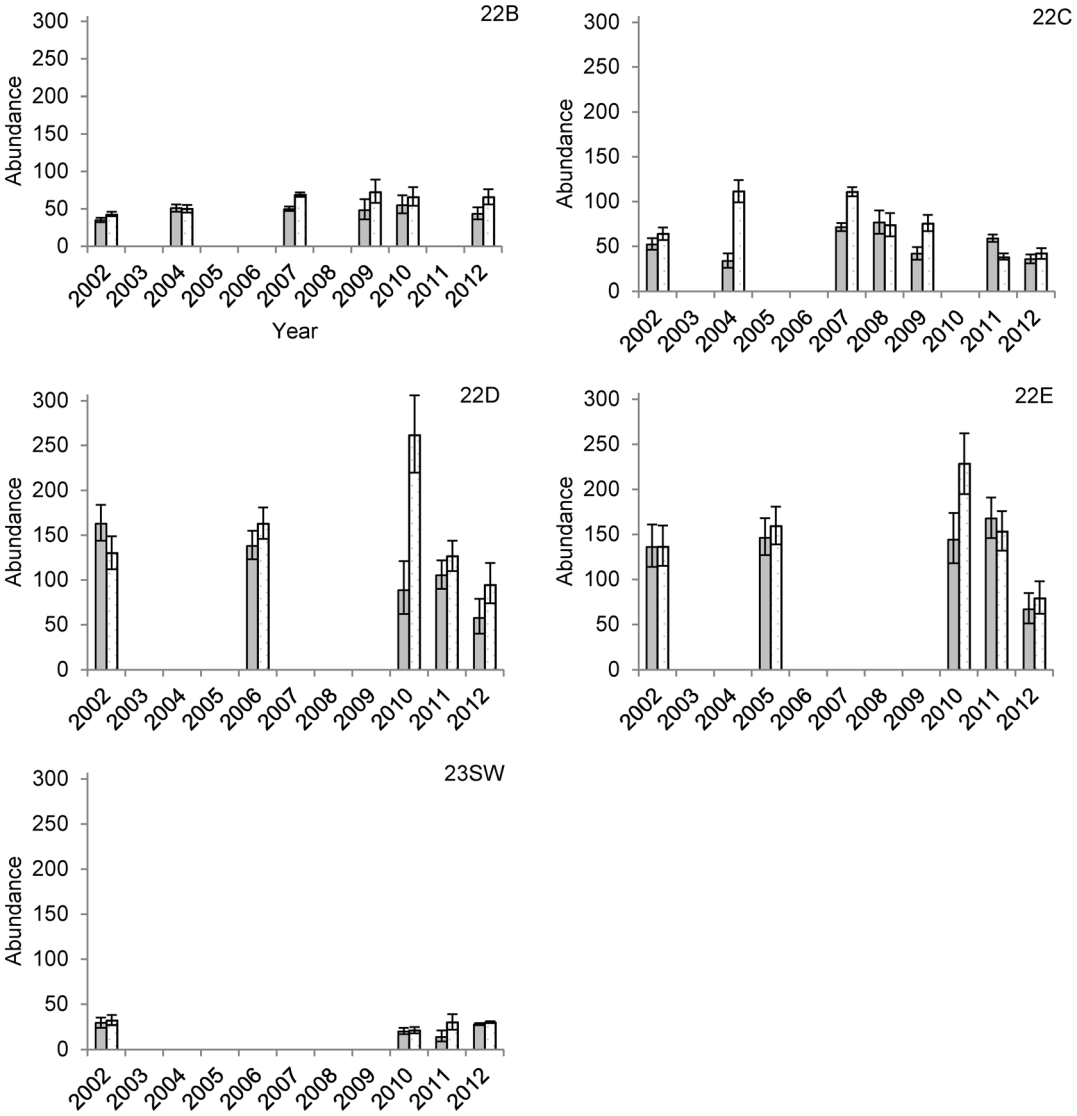
Estimated number of short-yearling and mature bull muskoxen on the Seward Peninsula, Alaska from 2002–2012. Estimated number of short-yearlings (gray bars) and mature bulls (stippled bars) present in 5 Game Management Subunits (GMSUs) on the Seward Peninsula, Alaska, USA from 2002 to 2012. Missing bars indicate years when composition surveys were not completed in a given GMSU. Error bars represent 95% Bayesian credible intervals.

**Figure 4:**
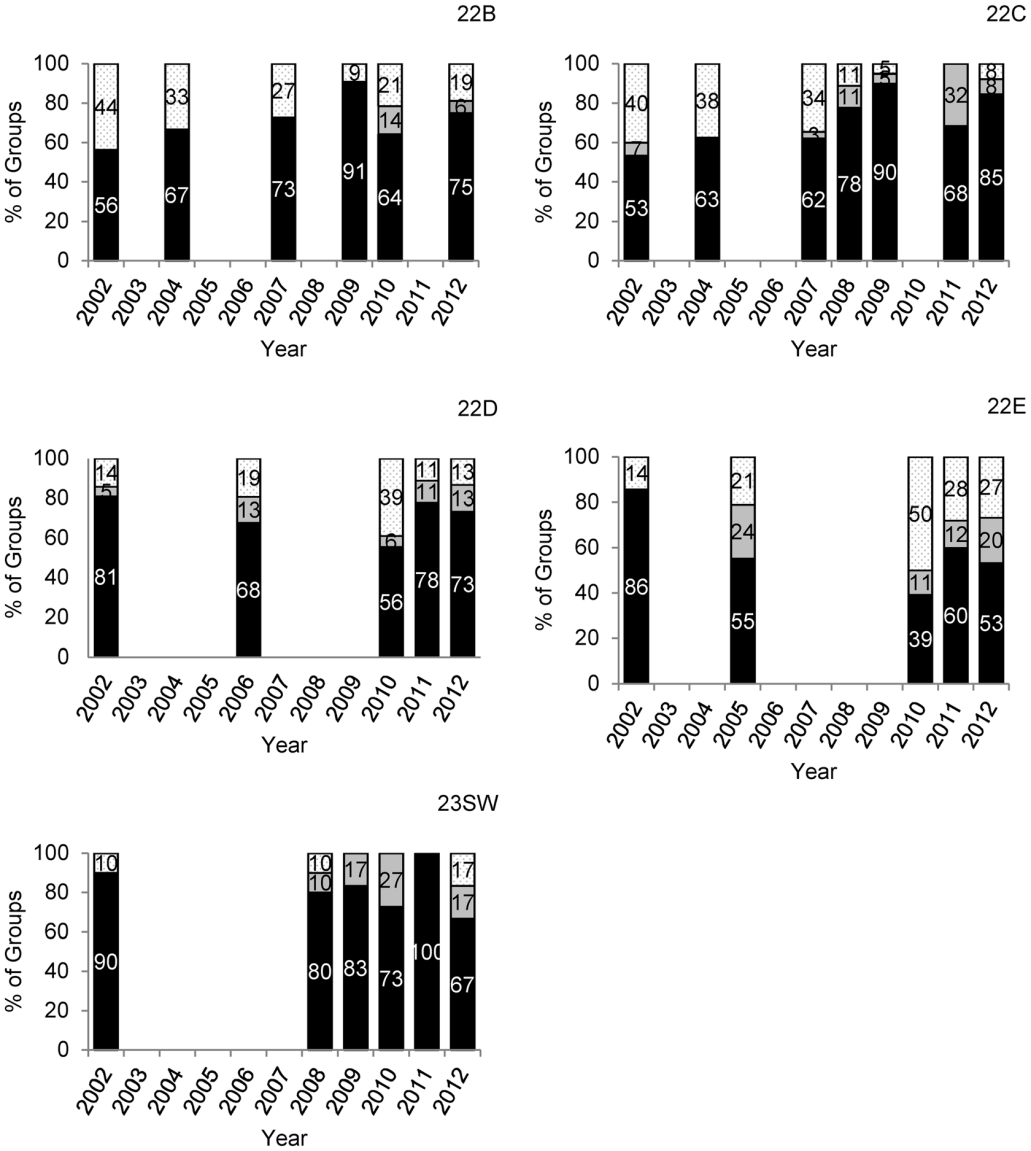
Proportion of muskox groups containing mature bulls on the Seward Peninsula, Alaska. Proportion of groups observed in 5 Game Management Subunits during the composition surveys containing mature bulls with other sex and age classes (black), no mature bulls (gray), and bulls only (stippled) on the Seward Peninsula, Alaska, USA from 2002 to 2012.

The numbers of muskoxen observed in the SPP study area increased through 2007, but the final two surveys suggested overall population growth had stopped by 2010 and then declined at a rate of -14%/year through 2012 (Table 1). Between 2010 and 2012, the estimated number of animals in 22C, 22D, and 22E (together containing approximately 70–80% of the total population) declined by 28%, 28%, and 51%, respectively, although numbers remained relatively unchanged in the remaining units (Table 1). We found that mature bull:mature cow ratios declined substantially in GMSUs 22B, 22C, and 23SW during the course of the study, while remaining relatively stable in 22D and 22E until after 2010 when ratios in these areas also appeared to decline (Table 2, Fig. 2). Short-yearling:mature cow ratios were more variable through time but declined in all GMSUs except 22E during the study period (Table 2, Fig. 3). Declines in mature bull:mature cow ratios were most severe in the most road accessible GMSU, 22C, where ratios changed at a rate of −12% (95%CI: −14% to −10%) annually. During this period, the proportion of bachelor groups also tended to decline, while the number of observed groups lacking mature bulls increased (Fig. 4). The average size of bachelor groups did not increase, rather the number of these groups generally declined through time accounting for the changes in proportions.

**Table 1:** Muskoxen counts by Game Management Sub-Unit.

		Game Management Unit				
Year	22B	22C	22D	22E	23SW	Total
2000	159	148	774	461	255	1797
2002	189	257	771	632	201	2050
2005	326	220	796	863	182	2387
2007	329	445	746	949	219	2688
2010^a^	420	402	878	879	175	2754
	(362–520)	(357–463)	(800–993)	(802–996)	(137–244)	(2561–3105)
2012^a^	460	289	629	431	222	2031
	(392–577)	(246–355)	(551–761)	(363–551)	(171–320)	(1806–2422)

a Values for 2010 and 2012 are the point estimates generated using distance sampling methods with 95% credible intervals shown in parentheses. Muskoxen counts by Game Management Sub-Unit for the 6 years during which all sub-units were surveyed between 2000 and 2012 on the Seward Peninsula, Alaska, USA.

**Table 2:** Estimated annual rates of change in sex and age ratios, mature bull abundance, and short-yearling abundance.

GMSU	λ^B:C^	λ^Y:C^	λ^B^	λ^Y^
22B	**−0.06**	**−0.08**	0.05	0.02
	(−0.8, −0.04)	(−0.10, −0.06)	(0.03, 0.06)	(0.00, 0.04)
22C	**−0.12**	**−0.07**	**−0.06**	0.00
	(−0.14, −0.10)	(−0.9, −0.05)	(−0.07, −0.05)	(−0.01, 0.01)
22D	−0.01	**−0.09**	0.00	**−0.08**
	(−0.03, 0.01)	(−0.11, −0.07)	(−0.01, 0.02)	(−0.09, −0.06)
22E	0.00	−0.01	−0.01	**−0.02**
	(−0.03, 0.03)	(−0.04, 0.02)	(−0.02, 0.01)	(−0.04, 0.00)
23SW	**−0.04**	**−0.06**	−0.01	**−0.03**
	(−0.07, −0.02)	(−0.09, −0.03)	(−0.04, 0.01)	(−0.06, −0.01)

Estimates of annual rates of change (λ) in mature bull:mature cow (B:C) and short-yearling:mature cow (Y:C) ratios and mature bull (B) and short-yearling (Y) abundance between 2002 and 2012 for 5 Game Management Subunits (GMSU) on the Seward Peninsula, Alaska, USA. Numbers in parentheses represent 95% confidence intervals. Bold numbers indicate estimated declines that do not include 0 in the 95% confidence interval.

Temporal patterns in the estimated total number of short-yearlings and mature bulls in each GMSU differed from patterns in composition. In 22C where total harvest exceeded 6% of the estimated population in consecutive years, the number of mature bulls declined throughout the study period (Table 2). In the remaining areas, mature bull numbers were relatively steady until after 2010 (Fig. 3). Short-yearling abundance declined in 22D, 22E, and 23SW throughout the study, and declined in most of the remaining GMSUs between 2010 and 2012 (Table 2, Fig. 3). The total number of bulls harvested generally increased in all GMSUs throughout the study (except for 2011), particularly between 2007 and 2010 (Table 3). However, the realized harvest rate of bulls increased dramatically, approaching half of the estimated number of bulls in some GMSUs (Table 3). The range of realized harvest rates on this segment of the population was lower in 22D and 22E and did not exceed 25% (Table 3), at least in years with corresponding composition data. In 2010, a sample of horns (*n* = 42) from harvested animals inspected by the ADFG indicated that 88% of the bulls harvested were mature animals in that year, despite the observed reductions in mature bull:mature cow ratios. In addition, we found that <12% of groups were likely missed during a given survey under the restricted conditions of the distance sampling protocol.

**Table 3:** Reported harvest and realized harvest rates of mature bull muskoxen.

			Game Management Subunit									
Year	22B		22C		22D		22E		23SW		Total	
	#	Rate	#	Rate	#	Rate	#	Rate	#	Rate	#	Rate
2000–2001	0		0		22		16		5		43	2.4%
2001–2002	6		2		23		9		5		45	
2002–2003	6	14%	5	8%	24	18%	18	13%	6	19%	59	2.9%
2003–2004	3		5		24		17		3		52	
2004–2005	7	14%	4	4%	17		34		5		67	
2005–2006	10		5		7		30	19%	0		52	2.2%
2006–2007	17		18		30	18%	19		3		87	
2007–2008	22	32%	25	23%	33		39		10		129	4.8%
2008–2009	9		30	41%	31		35		15		120	
2009–2010	14	19%	31	41%	34		42		13		134	
2010–2011	28	43%	24		49	19%	22	10%	4	19%	127	4.6%
2011–2012	17		1	3%	30	24%	28	18%	6	20%	82	
Total	139		150		324		309		75		997	

Reported number of bull muskoxen harvested (#) and estimated realized harvest rates (Rate = [number harvested/estimated number available] X 100) in each Game Management Subunit on the Seward Peninsula, Alaska, USA between regulatory years (i.e., July1 of the current year-June 30 of the following year) 2000 and 2011. Missing harvest rate values indicate years without appropriate composition or abundance data.

### Population Growth Relative to Harvest

**Figure 5:**
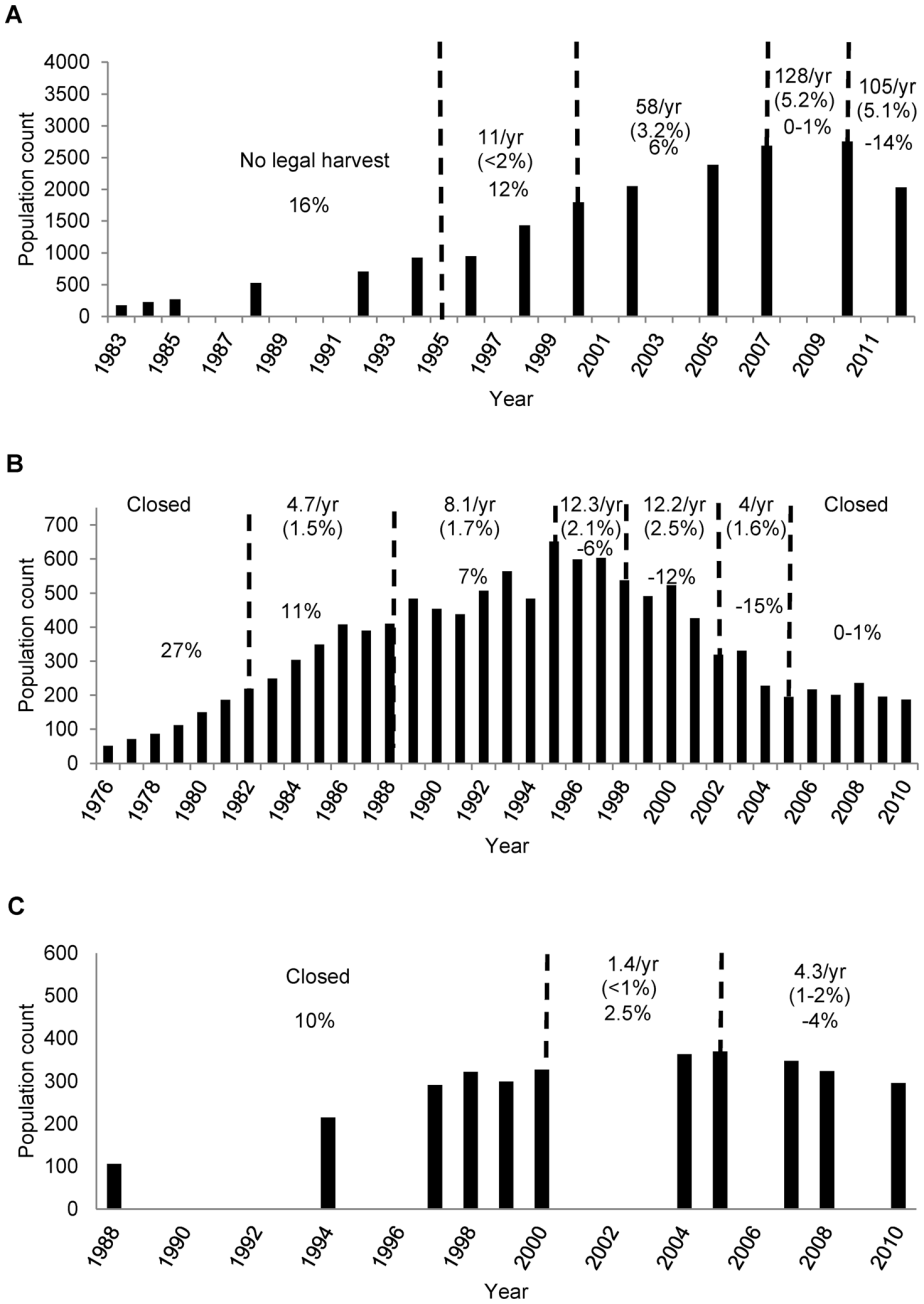
Population counts and harvest levels for the 3 mainland populations of muskoxen in Alaska. Population counts for the Seward Peninsula (A), Northeastern (B), and Cape Thomson (C) muskox populations in Alaska, USA. Dashed lines delineate periods with substantial changes in harvest. Values indicate the average number of bulls harvested annually during each period, the average annual overall harvest rate as a proportion of the total population (in parentheses), and the exponential rate of growth during each period. Data sources: Seward Peninsula [this study,31], Northeastern [33], [35], Cape Thompson [32], [47].

Annual rates of population growth for the SPP decreased disproportionately as harvest rates increased (Fig. 5A). After the onset of a small harvest averaging <2% of the population, population growth appeared to slow. Overall, annual harvest increased to an average of approximately 3% between 2000 and 2007, and the rate of population growth decreased by about 50% over the same period. At harvest rates of approximately 5% starting in 2007, growth was negligible through 2010 and then declined precipitously (14%/year) through 2012. Changes in harvest regimes appeared to be associated with decreases in population growth rate in the NEP as well, although harvest rates were lower and changes in growth occurred over a longer time frame (Fig. 5B). The average population growth rate was approximately 60–70% lower in the period after the onset of a 1.5–1.7% average harvest rate in the early 1980s, and the population declined dramatically after 1995 under an annual harvest of approximately 2% of the population. The population stabilized after harvest ended in 2006 and has remained stable (Fig. 5B). We identified a similar association between harvest rates and population growth in the CTP (Fig. 5C). Prior to the first harvest in 2000, the population grew at an annual rate of approximately 10%. Between 2000 and 2005 the average harvest rate was <1%, and population growth slowed to an average exponential rate of 2.5% annually. Between 2005 and 2010 the average annual harvest increased to 1–2% and the population declined at a rate of 4.5% annually. Although the rates of both population growth and harvest differed among the populations, the basic pattern of disproportionate decreases in growth after the implementation of increased harvest levels was consistent.

## Discussion

Our estimates of changes in abundance, sex and age ratios, and population growth rates through time coincided with increases in harvest rates, in agreement with our hypothesis that harvest of mature bulls may have secondary population-level effects in this species. While we were unable to rule out other potential causes such as changes in predator densities or density dependent effects, the observed relationship between high rates of harvest and changes in populations suggests that male-biased harvest regimes deserve careful consideration as potential driver of muskox populations. In the SPP, we found that population growth slowed, mature bull:mature cow and short-yearling:mature cow ratios declined, the number of bachelor groups declined, and the presence of mature bulls in mixed-sex groups declined in most GMSUs under increased harvest pressure. These population-level changes corresponded with increases in realized harvest rate estimates which suggested that in some years >40% of mature bulls may have been harvested in some GMSUs. Similar declines were also observed in the CTP and NEP under increasing harvest rates. Although our data could not be used to determine causation, when viewed in the context of the life-history characteristics of muskoxen, these patterns suggest that the harvest of mature bulls should be reduced until further research can identify the ultimate cause of observed declines in these populations.

Selective harvest regimes are expected to result in reduced mature bull:mature cow ratios [2], but the strong declines we observed in portions of the SPP (i.e., up to 70% over 10 years) likely indicated harvest levels were unsustainable. Past work on muskoxen has also suggested that high removal rates of mature bulls can lead to reductions in recruitment, possibly compounding the effect of harvest longer term. For example, Smith [48] observed declines in reproductive output concurrent with a selective males-only harvest regime in a predator-free system (i.e., Nunivak Island). In a portion of the NEP, Reynolds [33] documented declines in average calf production from 87 calves:100 cows prior to the implementation of harvest, to 61:100 between 1982 and 1986, and 38:100 between 1991 and 1996. Calf recruitment appeared to be lower in the CTP in later years as well, coincident with increases in harvest [32], [37], [47]. We found comparable declines in yearling:mature cow ratios in the SPP during the decade of this study. While the available data from these areas could not be used to establish harvest as the cause for observed declines, a consistent pattern of declining recruitment after the implementation of harvest does suggest that the selective harvest of mature males may be related to reductions in recruitment in this species.

Potential mechanistic explanations for decreased recruitment include: delayed birth dates, reduced birth synchrony, lowered calf body mass, and reduced pregnancy rates [2], [7], [8], [10], [14]. Pregnancy rates for mature cows in the SPP appeared to be quite high in recent years (>90% T. Gorn, unpublished data), suggesting that decreased calf survival may have been the ultimate cause of declining recruitment. Young male muskoxen may be less effective at maintaining a harem [48], and the presence of prime-aged bulls can synchronize estrus in females [49], [50]. Therefore, although the typical muskoxen calving season extends over several weeks, a reduction in the number of prime-aged bulls in the population could delay or prolong the calving period. In Alaskan ungulate populations, other studies have found decreased survival rates for calves born later in the season [51], [52] or outside of the peak calving period [53], suggesting that such delays could decrease calf survival in muskoxen as well. While these mechanisms have the potential to negatively affect recruitment, numbers of prime-aged bulls may expose all group members, and calves in particular, to higher levels of predation by decreasing the effectiveness of the group predator defense mechanism.

Wolves (*Canis lupus*) were traditionally considered to be the principal predator of muskoxen, and predation by grizzly bears (*Ursus arctos horribilis*) was considered rare [26], [54], [55]. The details of the defensive behavior of muskoxen are not fully understood, but it appears clear that prime-aged bulls in mixed-sex groups play a primary role in group defense [15], [17], [24], [25], [26] and they will aggressively defend themselves against grizzly bears [56]. A reduction in the effectiveness of group defense as numbers of prime-aged bulls are reduced through harvest offers one possible explanation for the increased instances of grizzly bear predation [56] and declines in calf production [33] observed in the NEP. Upon the elimination of harvest, the precipitous decline in the NEP ceased almost immediately, further supporting a possible link between the two. Grizzly bears are generally emerging from their dens during the calving season when other food sources are limited, and other work has found groups lacking mature bulls to be more nervous and flighty [21], [57]. Because muskoxen cannot easily outrun predators, individual animals and calves in particular are much more vulnerable to predation if the defensive approach is abandoned. Prior to the onset of harvest, bear predation was considered to be a rare occurrence on the Seward Peninsula, despite bears being common [58]. However, recent observations have indicated that bear predation has increased in the area, possibly explaining an adult cow mortality rate approaching 20% annually in some areas [31]. A lack of predator density estimates prevented us from evaluating the influence of the number and distribution of predators on muskox population trajectories, although we suspected that increases in successful predation attempts due to reduced numbers of prime-aged bulls could explain the disproportionate reductions in population growth and recruitment we and others have observed.

Other potential factors that could contribute to large-scale population declines include severe winter weather, large scale emigration from the study areas, or density dependent population limitation. Harsh winters with deep snow and icing events can reduce survival and recruitment and may be the primary factor limiting muskox populations in some areas [33], [59], [60]. However, in a separate study on the SPP, all observed non-human caused mortalities of radio-collared individuals occurred during spring and summer [31], supporting our assertion that severe winter weather conditions were likely not the immediate cause of death. This time period also corresponds to the interval when bears are not hibernating. Emigration has been observed in all 3 mainland populations [31], [32], [35], but the survey areas were very large, and in the case of the SPP is surrounded by the ocean on 3 sides, limiting the potential for extensive undocumented emigration. Recent surveys adjacent to our SPP study area to the east indicated a small, slowly growing population [31], confirming that emigration to adjacent areas was not driving large changes in population growth in the SPP. In muskoxen, reproductive rates are largely attributed to nutritional condition [61], [62], [63], females can give birth at age 2, and will calve in successive years under very favorable nutritional conditions [26], [63]. Preliminary data suggested that annual pregnancy rates were high in the SPP (>90% T. Gorn, unpublished data) and CTP, and a proportion of 2 yr old females were pregnant each year in both areas (L. Adams, unpublished data, J. Berger et al. unpublished data). If density-dependent limitations were influencing these populations, lower pregnancy rates, longer reproductive intervals, and later age of first reproduction would be expected. While we were unable to rule out density dependent effects, the available information provided little evidence that changes at the population level were due to population densities in any of the populations we considered.

Although our results indicate that declines in population size and mature bull:mature cow and short-yearling:mature cow ratios coincided with higher harvest, the nature of the available data complicated interpretation. Harvest regimes were established and composition surveys were conducted at the level of the subunit, potentially obscuring changes in subunits with lower numbers of animals (e.g., 22B or 23SW) through small-scale movements of a few groups from larger adjacent units (e.g., 22E). It is possible that changes in these subunits were buffered by small-scale immigration from the larger adjacent units, however, the overall pattern of population decline and declines in ratios was clear. In addition, the realized harvest rate differed through time in each subunit with some areas like 22C reaching higher levels of harvest prior to other areas (e.g., 22D, 22E). We expect that lower realized harvest rates in the early years of our project explain the later timing of declines in abundance and sex/age ratios in subunits 22D and 22E. The lack of parallel declines in abundance and composition throughout the study for all subunits may reflect these differences in timing rather than indicating differing responses among subunits. If our interpretation is correct, these differences suggest that there may be a lag between the timing of the implementation of increased harvest and observed changes in abundance and composition.

We acknowledge that our data do not provide a definitive explanation for the observed population declines and multiple factors may play important roles. However, the available evidence does suggest that the selective harvest of mature bull muskoxen should be considered as a potential cause of observed declines in recruitment and population growth. While we were unable to exclude influences of density dependence or changes in predator densities as primary drivers of population change, our results suggest harvest could be an important driver. We suspect that the overall reduction in the number and average age of bulls in each population may have increased the opportunity for predation on cows and calves (particularly by bears), although this hypothesis will require further testing. Predation pressure may be particularly high in the spring when grizzly bears first emerge from dens and muskox groups that have experienced harvest are least likely to contain mature bulls. A concurrent decline in recruitment and possibly cow survival, if related to mature bull abundance, could explain the dramatic and sustained decline (approximately 60% between the mid-1990s and mid-2000s) in the NEP, the recent 28% decrease in the SPP between 2010 and 2012, and the approximately 20% decrease in the CTP between 2005 and 2010. If our suspicions are correct, the low numbers of large bulls and associated bachelor groups due to years of poor recruitment may help explain the failure of the NEP to recover after the cessation of harvest [35], and the consistent declines in the SPP and CTP. Until appropriate data are available to establish the cause of population declines in harvested muskox populations, our results suggest that managers should consider the potential importance of prime-aged bulls to overall population productivity and growth, and future conservation and harvest programs should be structured accordingly.

### Conclusions

After examining the available data, we propose that male-biased harvest rates based on total population size may be inappropriate for muskoxen. With observed concurrent declines in short-yearling:mature cow and mature bull:mature cow ratios, as well as the overall population in the SPP, we recommend that annual harvest be restricted to <10% of the estimated number of mature bulls in the interest of conservation. Reasonable rates might be lower, particularly following years with poor recruitment or in declining populations, and the elimination of harvest should be considered if mature bull:mature cow ratios fall below approximately 20:100. If our hypothesis is correct, we suspect that higher harvests and positive population growth rates may be sustainable in the future if sex ratios were returned to near pre-hunt levels (>50–70 mature bulls:100 mature cows). A formal adaptive management framework could provide a mechanism for assessing the relationship between harvest and population trajectories and may reduce the risk of unsustainable harvest rates in the future [64], [65], [66]. For the SPP, we suggest range-wide abundance and composition surveys be conducted sequentially (within a year) every other year to best monitor the continuing effects of harvest on population structure and trajectory. Current effort for the composition surveys (i.e., ≥15 groups or 200 individuals per GMU) appears to be adequate, but if more detailed information is needed for specific areas, larger samples may be necessary. Further research focused on pregnancy rates, body condition, timing and causes of mortality, predation rates and predator densities, and comparisons of survival and recruitment rates of harvested vs. unharvested sub-populations will be necessary to establish the causal mechanism for population declines in harvested populations.
